# A comparison of synthetic data generation and federated analysis for enabling international evaluations of cardiovascular health

**DOI:** 10.1038/s41598-023-38457-3

**Published:** 2023-07-17

**Authors:** Zahra Azizi, Simon Lindner, Yumika Shiba, Valeria Raparelli, Colleen M. Norris, Karolina Kublickiene, Maria Trinidad Herrero, Alexandra Kautzky-Willer, Peter Klimek, Teresa Gisinger, Louise Pilote, Khaled El Emam

**Affiliations:** 1 Centre for Outcomes Research and Evaluation, Research Institute of the McGill University Health Centre, 5252 De Maisonneuve Blvd, Office 2B.39, Montréal, QC H4A 3S5 Canada; 2 Department of Internal Medicine III, Division of Endocrinology and Metabolism, Gender Medicine Unit, Medical University of Vienna, Vienna, Austria; 3 Faculty of Medicine, McGill University, Montreal, Canada; 4 Department of Translational Medicine, University of Ferrara, Ferrara, Italy; 5 Faculty of Nursing, University of Alberta, Edmonton, AB Canada; 6 Heart and Stroke Strategic Clinical Networks, Alberta Health Services, Alberta, Canada; 7 Karolinska Institute, Stockholm, Sweden; 8 Clinical & Experimental Neuroscience (NiCE-IMIB-IUIE), School of Medicine, University of Murcia, Murcia, Spain; 9 Section for Science of Complex Systems, CeMSIIS, Medical University of Vienna, Vienna, Austria; 10 Complexity Science Hub Vienna, Vienna, Austria; 11 Division of Endocrinology and Metabolism, Medical University of Vienna, Vienna, Austria; 12 Divisions of Clinical Epidemiology and General Internal Medicine, McGill University Health Centre Research Institute, Montreal, QC Canada; 13 Children’s Hospital of Eastern Ontario Research Institute, 401 Smyth Road, Ottawa, ON K1H 8L1 Canada; 14 School of Epidemiology and Public Health, University of Ottawa, Ottawa, ON Canada; 15 Replica Analytics Ltd, Ottawa, ON Canada

**Keywords:** Cardiology, Medical research, Engineering

## Abstract

Sharing health data for research purposes across international jurisdictions has been a challenge due to privacy concerns. Two privacy enhancing technologies that can enable such sharing are synthetic data generation (SDG) and federated analysis, but their relative strengths and weaknesses have not been evaluated thus far. In this study we compared SDG with federated analysis to enable such international comparative studies. The objective of the analysis was to assess country-level differences in the role of sex on cardiovascular health (CVH) using a pooled dataset of Canadian and Austrian individuals. The Canadian data was synthesized and sent to the Austrian team for analysis. The utility of the pooled (synthetic Canadian + real Austrian) dataset was evaluated by comparing the regression results from the two approaches. The privacy of the Canadian synthetic data was assessed using a membership disclosure test which showed an F1 score of 0.001, indicating low privacy risk. The outcome variable of interest was CVH, calculated through a modified CANHEART index. The main and interaction effect parameter estimates of the federated and pooled analyses were consistent and directionally the same. It took approximately one month to set up the synthetic data generation platform and generate the synthetic data, whereas it took over 1.5 years to set up the federated analysis system. Synthetic data generation can be an efficient and effective tool for enabling multi-jurisdictional studies while addressing privacy concerns.

## Introduction

Cardiovascular diseases (CVD) continue to represent the leading cause of mortality and morbidity amongst women and men worldwide^1^. Biological differences between the sexes such as anatomical and physiological variations in coronary arteries and the autonomic nervous system alter the development and progression of CVD^2^. However, the environment and lifestyle^3^ as well as individuals’ identity, roles, and relations in society may play an important role. These characteristics are gendered in a way that they affect males and females differently and evolve through early life to adulthood^4^. The effect of these gendered factors can vary between countries with different cultural and political biases^5^. Investigating the impact of sex on CVD across multiple countries requires pooling data sourced from these jurisdictions.

However, sharing and pooling health data across institutions and across national and international jurisdictions has been a challenge^6^. Privacy concerns are key barriers to data sharing and data access^7,8^, particularly in EEA countries where the General Data Protection Regulation (GDPR) imposes high standards for data sharing that are often difficult to meet in practice^9,10^. This raises a particular challenge given that the GDPR is serving as a template regulation around the globe^10^.

One approach to address such privacy concerns has been to perform a distributed data analysis whereby the analysis is performed within each dataset locally and then final results combined through a meta-analysis. As an example, in a study evaluating the effectiveness of different statins in each of 3 Canadian provinces^11^, the hazard ratios for different statins were combined using a fixed-effects model, with weight being the inverse of the variance of the province-specific parameter estimate^12^. However, because the same analysis needs to be executed multiple times by different teams in each province, this general approach has not resulted in timely results in practice^13^.

Another option which can enable the timely sharing of datasets in a privacy protective manner is synthetic data generation (SDG)^14,15^. There have been multiple synthetic health data releases in the US^16,17^, the UK^18–20^ and other European countries^21,22^. None of these efforts pooled datasets across jurisdictions to enable cross-country analysis.

In this study, we evaluate whether SDG can be applied for pooling data to enable international comparative studies. Our objective was to assess country differences of the effect of sex on the cardiovascular health (CVH) of Canadian and Austrian populations. The datasets used in this study were from the Canadian Community Health Survey (CCHS) that was administered in 2014 in Canada (n = 63,522), and the Austria Health Interview Survey (ATHIS 2014, n = 15,771) which was conducted as part of the European Health Interview Survey series. These surveys collect information on health status, psychosocial factors, and healthcare resource utilization.

**Figure 1:**
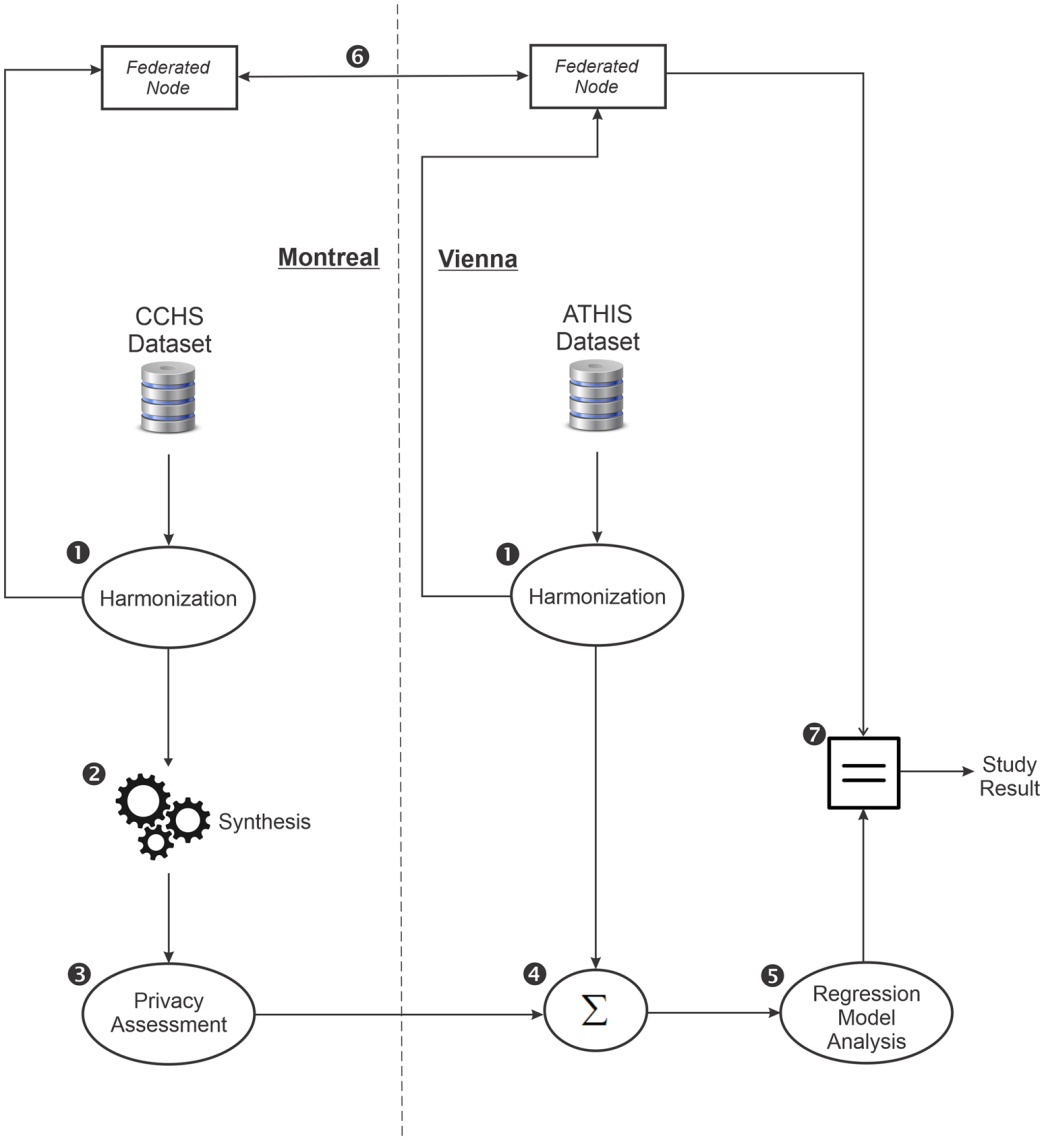
The data synthesis and federated analysis workflow.

The Canadian data was synthesized and sent to Austria to be pooled with the original Austrian dataset, and a multivariable regression model was constructed from the pooled dataset. To generate synthetic data, we used sequential classification and regression trees^23,24^. The results were compared to the ground truth results obtained through a federated analysis on the source data. The federated analysis was performed using the DataSHIELD method and tools^25^. The DataSHIELD approach exchanges the intermediate results among the nodes which means that its analysis gives the same the results as those obtained from pooling the original datasets. The study workflow is shown in Fig. 1.

Starting from relatively similar points with the availability of robust software to perform federated analysis and SDG, and a relatively good knowledge of privacy enhancing technologies within the team, the federated analysis took eighteen months to set up operationally and obtain results, whereas the SDG approach took in total one month to set up, install, and execute. Therefore, testing whether an SDG method can produce the same results as those obtained from source data and if such an approach can be privacy protective, could enable significantly more efficient pooling of data across jurisdictions.

## Results

### Privacy risks of synthetic data

The privacy of the synthetic CCHS data was assessed using a membership disclosure test (step 3 in Fig. 1). Membership disclosure risk assessment is a common way to evaluate the privacy risks in synthetic datasets^26–29^, and is defined as an adversary, using the information in synthetic data, determines that a real target person was included in the original dataset used as input for synthetic data generation (i.e. was a member of the training dataset). Knowing that an individual was in the training data can reveal sensitive attributes about that individual.

The relative membership disclosure F1 score^30^ was 0.001, indicating that the ability for an adversary to predict membership is quite poor. The low value means that the synthetic Canadian dataset can be deemed as having low disclosure risks.

### Descriptive statistics

The CCHS cycle 2014 included 55.3% females, while the ATHIS Cycle 2014 included 55.7% females (Table 1). The Austrian participants were slightly younger than the Canadians. However, there was an age difference between males and females in the Canadian participants with slightly older females (p < 0.001) but similar in the Austrian participants (p = 0.32). There was a small difference in hypertension between males and females in the Canadian dataset (M vs. F: 24.2% vs. 25.1%), and in the Austrian dataset (M vs F: 21.4% vs. 18.9%). In the Austrian dataset there were more females that were immigrants (M vs F: 7.6% vs. 9.6%) compared to the Canadian dataset where there was no difference in immigration status (M vs. F: 14.5% vs. 14.4%). Otherwise, the two datasets were similar in terms of male vs female comparisons with the following patterns: more females had a lower BMI, more males had diabetes and were smokers, more females were divorced or widowed, more females lived in single occupant households, and more females lived in low- or medium-income households.

**Table 1:** Comparison of baseline characteristics for the Canadian and Austrian datasets.

Baseline characteristics, %	CCHS-source				ATHIS-source			
	Overall	Male	Female	p-value	Overall	Male	Female	p-value
Age	N = 63,522	N = 28,408	N = 35,114		N = 15,671	N = 6,950	N = 8,721	
< 20	10.2	11.8	8.9	< 0.001	3.5	3.9	3.3	0.32
20–29	10.5	10.9	10.1		12.1	12.0	12.2	
30–39	11.2	11.0	11.3		16.3	16.3	16.3	
40–49	10.4	10.9	9.9		22.2	21.8	22.5	
50–59	17	16.9	17.1		22.2	22.1	22.4	
60–69	20	19.7	20.2		14.1	14	14.1	
> = 70	20.8	18.7	22.5		9.4	9.8	9.2	
BMI	N = 59,244	N = 26,922	N = 32,322		N = 15,771	N = 6,985	N = 8,786	
< 25	45.4	39.2	50.6	< 0.001	53.3	42.8	61.6	< 0.001
HX Hypertension	N = 63,306	N = 28,289	N = 35,017		N = 15,771	N = 6,985	N = 8,786	
	24.7	24.2	25.1	0.004	20.0	21.4	18.9	< 0.001
HX Diabetes	N = 63,435	N = 28,375	N = 35,060		N = 15,771	N = 6,985	N = 8,786	
	9.3	10.3	8.5	< 0.001	4.3	5.1	3.7	< 0.001
HX smoking	N = 62,969	N = 28,154	N = 34,815		N = 12,225	N = 5,282	N = 6,943	
	18.1	20.3	16.3	< 0.001	36.9	44.0	31.5	< 0.001
Marital status	N = 63,392	N = 28,350	N = 35,042		N = 15,771	N = 6,985	N = 8,786	
Single	28.1	32.2	24.7	< 0.001	30.3	33.4	27.8	< 0.001
Divorced/widowed	20.9	13.7	26.8		15	10.0	19.0	
Common-law/married	51	54.1	48.4		54.7	56.6	53.2	
Household size	N = 63,484	N = 28,394	N = 35,090		N = 15,771	N = 6,985	N = 8,786	
1	27.8	23.3	31.4	< 0.001	15	13.2	16.4	< 0.001
2	39.4	41.8	37.5		37.1	38.1	36.4	
3	13.1	14.0	12.3		20.7	20.9	20.4	
4	12.9	13.6	12.3		18.7	19.1	18.3	
5 & 5 +	6.9	7.3	6.5		8.6	8.7	8.5	
Education	N = 62,501	N = 27,919	N = 34,582		N = 15,771	N = 6,985	N = 8,786	
< Secondary	24.1	25.2	23.2	< 0.001	15.6	10.6	19.6	< 0.001
Secondary	19.6	18.8	20.3		52.1	54.7	50	
Post secondary	4.7	4.8	4.5		20.0	20.3	19.7	
> Post secondary	51.6	51.2	52.0		12.3	14.3	10.7	
Household Income	N = 63,456	n = 28,373	n = 35,083		N = 15,771	n = 6,985	n = 8,786	
Low	10.2	7.7	12.3	< 0.001	36.0	32.2	39.0	< 0.001
Medium	39.7	36.9	42.1		21.1	20.3	21.7	
High	50.1	55.4	45.6		42.9	47.5	39.3	
Immigrant	N = 61,471	N = 27,501	N = 33,970		N = 15,771	N = 6,985	N = 8,786	
	14.4	14.5	14.4	0.61	8.7	7.6	9.6	< 0.001

N = Overall number of respondents for each variable within male or female sex categories.

p-value: The difference between male and female and was not Bonferroni corrected in this table.

### Comparison of pooled partially synthetic data and federated analysis results

#### Descriptive statistics

A comparison of the marginal distributions between males and females in Table 2 showed consistently similar results in the federated and pooled analyses of partially synthetic data across all variables, with the standardized mean differences (SMD) consistently below the 0.1 threshold^31^.

**Table 2:** Descriptive statistics for the federated and pooled analysis.

Baseline characteristics, %	Federated analysis				Pooled analysis average SMD		
	Overall	Male	Female	p-Value	Overall	Male	Female
Age	N = 78,734	N = 35,358	N = 43,376				
< 20	8.8	10.3	7.7	< 0.001	0.003	0.284	0.177
20–29	10.8	11.1	10.5				
30–39	12.2	12	12.3				
40–49	12.7	13.1	12.4				
50–59	18.1	17.9	18.1				
60–69	18.8	18.6	19				
> = 70	18.6	17	19.8				
Marital status	N = 79,163	N = 35,335	N = 43,828				
Single	28.5	32.5	25.3	< 0.001	0.003	0.011	0.007
Divorced/widowed	19.8	12.9	25.3				
Common-law/married	51.7	54.6	49.4				
Household size	N = 79,255	N = 35,379	N = 43,876				
1	25.2	21.3	28.4	< 0.001	0.015	0.021	0.015
2	38.9	41	37.3				
3	14.6	15.4	13.9				
4	14	14.7	13.5				
5 & 5 +	7.2	7.5	6.9				
Education	N = 78,272	N = 34,904	N = 43,368				
< Secondary	22.4	22.3	22.4	0.4	0.018	0.017	0.021
Secondary	26.2	26	26.3				
Post secondary	7.8	7.9	7.6				
> Post secondary	43.7	43.8	43.6				
Household income	N = 79,227	N = 35,358	N = 43,869				
Low	15.3	12.5	17.6	< 0.001	0.018	0.021	0.017
Medium	36	33.6	38				
High	48.6	53.9	44.4				
Immigrant	N = 77,242	N = 34,486	N = 42,756				
	13.3	13.1	13.4	0.2	0.001	0.002	0.005

N = Overall number of respondents for each variable within male or female sex categories.

P-values compared males vs females and was not Bonferroni corrected in this table.

SMD values were averaged across the ten pooled partially synthetic datasets, and compare the pooled with the equivalent federated dataset.

Males tended to be younger, there were more females with normal BMI (M vs. F: 39.9% vs. 52.9%), more males had diabetes (M vs. F: 9.2% vs. 7.5%) and were smokers (M vs. F: 24.1% vs. 18.8%). There were more males that were single (M vs. F: 32.5% vs. 25.3%), and males were more likely to be in a household with a high income (M vs. F: 53.9% vs. 44.4%). Females were more common in single-person households (M vs. F: 21.3 vs. 28.4%) and were more likely to be divorced or widowed (M vs. F: 12.9% vs. 25.3%). There were no significant differences between sexes on hypertension (M vs. F: 23.6% vs. 23.9%), post-secondary education and higher (M vs. F: 51.7% vs. 51.2%), and whether the individual was an immigrant (M vs. F: 13.1% vs. 13.4%).

#### Determinants of cardiovascular health: univariable analysis

The outcome variable of interest was CVH calculated through a modified CANHEART index in both countries^32^. Overall, 70.7% of Canadians and 67.9% of Austrians had a CANHEART score greater than three.

Table 3 shows the parameter estimates, confidence intervals, and p-values for the pooled and federated univariable regression analysis. The results were similar between the two methods of analysis, with the substantive conclusions being the same from both approaches.

**Table 3:** Univariable linear regression using the federated and pooled analysis.

CANHEART score**	Federated analysis	Pooled analysis
	Regression coeff***	Regression coeff***
Sex (*ref:* males)	0.19 (0.17, 0.20)*	
Marital status (Ref: single)		
Divorced/widowed	− 0.60 (− 0.62, − 0.58)*	− 0.61 (− 0.63, − 0.59)*
Common-law/married	− 0.40 (− 0.42, − 0.38)*	− 0.41 (− 0.43, − 0.40)*
Household size	0.18 (0.17, 0.19)*	0.19 (0.18, 0.19)*
Education	0.04 (0.03, 0.04)*	0.04 (0.03, 0.04)*
Household income (reverse coded)	− 0.18 (− 0.19, − 0.17)*	− 0.19 (− 0.20, − 0.18)*
Immigrant (ref: No)	0.09 (0.07, 0.11)*	0.09 (0.08, 0.12)*
Age	− 0.17 (− 0.18, − 0.17)*	− 0.17 (− 0.18, − 0.17)*
Country (ref: CA)	− 0.03 (− 0.04, − 0.01)*	− 0.04 (− 0.05, − 0.02)*

*p < 0.05.

**CANHEART index: A measure of CVH in the population, consisting of 4 cardiometabolic risk factors (i.e., smoking, obesity, diabetes and hypertension), 0 (worst) to 4 (ideal).

***Regression Coefficient: the degree of change in the CANHEART index for every 1-unit of change in the predictor variables.

Females had better CVH than males (pool vs. fed: 0.18 vs. 0.19), as well as individuals in larger households (pool vs. fed: 0.19 vs. 0.18) and immigrants (pool vs. fed: 0.09 vs. 0.09). Older individuals had worse CVH (pool vs. fed: − 0.17 vs. − 0.17), as well as divorced/widowed individuals (pool vs. fed: − 0.61 vs. − 0.6) and common-law/married individuals (pool vs. fed: − 0.41 vs. − 0.4) compared to single individuals. Lower income individuals also had worse CVH (pool vs. fed: − 0.19 vs. − 0.18). There was a weak positive relationship between higher education and CVH (pool vs. fed: 0.04 vs. 0.04). The weakest relationship was between country and CVH whereby the effect size was similar between federated analysis and pooled analysis (− 0.04 vs. − 0.03), indicating slightly worse CVH among the Austrian respondents.

#### Determinants of cardiovascular health across countries: interaction analyses

In the multivariable analysis of the main effects, the parameter estimates of the federated and pooled analysis were directionally the same as for the univariable analysis, and the comparison between the federated and pooled analysis yields the same conclusions as for the univariable analysis (see Table 4).

**Table 4:** Multivariable main effects models for predicting CVH in federated and pooled analyses.

CANHEART score**	Federated analysis	Pooled analysis
	Regression coeff ***	Regression coeff***
Sex (ref: male)	0.25 (0.23, 0.26)*	0.24 (0.23, 0.25)*
Education	0.04 (0.04, 0.05)*	0.04 (0.04, 0.05)*
Marital status (ref: Single)		
Divorced/widowed	− 0.12 (− 0.14, − 0.09)*	− 0.11 (− 0.14, − 0.09)*
Married	− 0.15 (− 0.17, − 0.13)*	− 0.16 (− 0.18, − 0.14)*
Household size	0.05 (0.04, 0.06)*	0.06 (0.05, 0.06)*
House income (reverse coded)	− 0.08 (− 0.09, − 0.07)*	− 0.09 (− 0.10, − 0.08)*
Immigrant(ref: No)	0.13 (0.12, 0.15)*	0.14 (0.13, 0.16)*
Age	− 0.13 (− 0.14, − 0.13)*	− 0.14 (− 0.14, − 0.13)*
Country (ref: CA)	− 0.01 (− 0.03, 0.002)	− 0.02 (− 0.04, 0.00)
R^2^	0.163	0.165

*p < 0.05.

**CANHEART index: A measure of CVH in the population, consisting of 4 cardiometabolic risk factors (i.e. smoking, obesity, diabetes and hypertension), 0 (worst) to 4 (ideal).

***Regression Coefficient: the degree of change in the CANHEART index for every 1-unit of change in the predictor variables.

In the multivariable analyses considering the country interactions to determine whether country moderates the relationship between the other variables and CVH, the impact of several factors differed between countries (Table 5). For example, although males in Austria have lower CVH than males in Canada, females in Austria had better CVH than females in Canada. Also, at lower levels of education, CVH was lower among the Austrian respondents, but this country difference changed as education levels increased whereby Austrians with high levels of education had higher CVH. At the highest level of education Austrians had better CVH than Canadians. Immigrants had better CVH in Canada compared to Austria, but worse CVH than non-immigrants in both countries.

**Table 5:** Multivariable model with country interactions for federated and pooled analysis.

CANHEART score**	Federated analysis		Pooled analysis	
	Main effect coefficient (95% CI)	Country interaction coefficient (95% CI)	Main effect coefficient (95% CI)	Country interaction coefficient (95% CI)
Sex (ref: male)	0.226 (0.211, 0.24)*	0.157 (0.122, 0.191)*	0.215 (0.201, 0.229)*	0.168 (0.134, 0.202)*
Education	0.036 (0.03, 0.042)*	0.08 (0.063, 0.101)*	0.035 (0.029, 0.040)*	0.084 (0.065, 0.103)*
Marital status (ref: Single)				
Divorced/widowed	− 0.112 (− 0.138, − 0.087)*	− 0.039 (− 0.103, 0.02)	− 0.104 (− 0.129, − 0.079)*	− 0.048 (− 0.112, 0.015)
Married	− 0.169 (− 0.19, − 0.147)*	0.057 (0.008, 0.107)*	− 0.166 (− 0.187, − 0.145)*	0.056(− 0.006, 0.105)
Household size	0.051 (0.042, 0.059)*	− 0.011 (− 0.02, 0.007)	0.05 (0.042, 0.058)*	− 0.011 (− 0.029, 0.007)
House income (reverse coded)	− 0.13 (− 0.145, − 0.12)*	0.12 (0.1, 0.147)*	− 0.124 (− 0.137, − 0.112)*	0.116 (0.094, 0.138)*
Immigrant (ref: No)	0.163 (0.143, 0.183)*	− 0.207 (− 0.265, − 0.15)*	0.166 (0.146, 0.186)*	− 0.210 (− 0.268, − 0.153)*
Age	− 0.126 (− 0.132, − 0.12)*	− 0.06 (− 0.077, − 0.048)*	− 0.128 (− 0.133, − 0.122)*	− 0.061 (− 0.075, − 0.047)*
Country (ref: CA)	− 0.22 (− 0.335, − 0.117)*		− 0.234 (− 0.343, − 0.126)*	
R^2^	0.168		0.170	

*p < 0.05.

There is one difference in the interaction parameters between the federated and pooled models. While the significance of the interaction parameter for being married differs between the two approaches, the substantive conclusions are the same in that being married has lower CVH in both countries, and CVH is lower in Austria than in Canada irrespective of marital status.

The effect size for the country variable is larger in the interaction model compared to the univariable model and main effects only multivariable models. The interaction model assumes a contingency effect of country and therefore the country parameter should not be interpreted by itself^33^.

#### Elapsed time comparisons

A significant time elapsed to set-up the necessary servers in multiple locations with the requisite security protocols for the federated analysis (these servers hold the original sensitive datasets and needed to be accessible remotely from a different jurisdiction, requiring the introduction of additional security protocols and checks), and to obtain the necessary approvals (Table 6). The programming required for DataSHIELD had to be done anew since common regression R packages used by the analysts were not usable in a federated context. Once the multiple nodes have been set up the processing speeds are comparable.

**Table 6:** The difference in elapsed time between the federated analysis and the pooled analysis.

	Synthetic data analysis		Federated analysis	
	Canada	Austria	Canada	Austria
Planning and coordination	1 month	1 month	1.5 years	1.5 years
Server set up time	< 1 h	< 1 h	10 h	8 h
Programming and coding/learning UI	1 day	1 day	3 weeks	–
Execution time	Seconds	Seconds	Minutes	–

These values demonstrate the advantage of synthetic data relatively speaking. An important context here is that the DataSHIELD system was being set up in two academic medical centers, which may have an impact on timing. Plus, this work was done during the COVID-19 pandemic which would have impacted the speed at which multi-institutional and multi-jurisdictional projects progressed.

## Discussion

### Summary

Our results highlight the country specific effects of sex on CVH and demonstrated slightly better CVH in Canadians compared with Austrians. Marital status, low household income and not being single were associated with worse CVH while female sex, greater household size, higher level of education, and being an immigrant were associated with better CVH in federated and pooled datasets. The magnitude of these factors differed between Austria and Canada.

The result of this secondary analysis of population-based datasets revealed that synthetic data generation methods using sequential classification and regression trees can be used to pool datasets across countries for international studies. The analytical conclusions were the same for the models developed using the pooled partially synthetic dataset as the ground truth model developed using federated analysis in various analytical steps including descriptive, univariable analysis and multivariable main effects and country interaction models. While previous observational studies have compared synthetic and real data^34–36^, there has been no population-based study testing the use of SDG for pooling datasets across jurisdictions and comparing it to a federated approach.

We provided evidence that synthetic data has similar utility compared to the ground truth generated through federated analysis. While there was one difference in regression model parameters, this was for a weak effect size. Where weak effects are important then the pooled partially synthetic data can be used for exploratory analysis to validate assumptions while procedures for the exchange of the original data are set up.

The significantly lower effort in getting to the results using synthetic data can enable researchers to efficiently share data across jurisdictions. Data synthesis was completed in approximately one month whereas it took eighteen months to set up the federated analysis system across two nodes. It is expected that further substantial work would be needed to set up additional nodes to accommodate the inclusion of other countries in the international analysis.

The use of synthetic data will allow merging a variety of population-based databases globally and across jurisdictions nationally and internationally. For our specific work, this would allow us to assess the association of sex with the cardiovascular health of populations while evaluating the effect of geo-politico-cultural differences in disease risk.

We found that being divorced, widowed, or married was associated with worse CVH compared to being single. Similar results were obtained in an analysis of data from the US, where single participants had better health habits and lower preventable risk factors than married/widowed or divorced in the National Health Interview Survey^37^. While singles might have better CVH, evidence for the mortality rate from CVD in single participants compared to married participants is still inconsistent^38–41^. Studies have identified the increased prevalence of non-traditional CVH risk factors including stress, depression, recreational drugs, and other socioeconomic risks in non-married groups that can indeed impact these subjects additionally^42^. This may explain the greater risk of CVD and mortality in non-married compared to married subjects in those studies. It is also reported that these acute stressors are even greater in those widowed and divorced (spousal death, divorce)^43^, which may strengthen the development of CVD compared to single and married in our study.

Lower socioeconomic status is associated with increased risk of CVD and mortality^3^. Our results are generally supportive demonstrating a positive effect of higher education. There was significant interaction between many covariates and country. Males in Austria have worse CVH than males in Canada. Also, at lower levels of education CVH is worse among the Austrian respondents, but this country specific effect reverses as education levels increase: at the highest level of education Austrians seem to have better CVH than Canadians. Moreover, immigrants have better CVH in Canada than Austria, and non-immigrants have better CVH overall that is also higher in Canada. Being married has worse CVH in both countries, and CVH is lower in Austria than in Canada across all values of marital status. These results suggest groups to be targeted for improving CVH are country specific.

### Limitations and future work

One of the limitations of our study is using only a single data synthesis method. Application of other types of data synthesis and comparing the utility of those methods with those from the current study is recommended in future studies. We only pooled two datasets. Multi-jurisdictional studies may pool datasets across more than two jurisdictions, and we did not test utility when multiple datasets are synthesized and pooled.

Other methods for privacy-reserving analysis of multi-jurisdictional data include performing a meta-analysis. However, because the same, potentially complex, analyses must be performed multiple times, the timelines of this approach has in practice proven to be challenging^13^. The use of synthetic data generation can help accelerate the time to results.

## Conclusions

Our results indicate high utility for the pooled partially synthetic dataset, and low privacy risks for the synthetic data, in addition to an elapsed time advantage when compared to the federated analysis platform. Our analysis identified factors with a differential effect on CVH depending on country where a person lives. Hence, interventions will need to be country specific.

## Methods

The objective of the analysis was to assess country-level differences in the role of sex on cardiovascular health (CVH) using a pooled dataset of Canadian and Austrian individuals.

### Datasets used

The CCHS and ATHIS variables/questions that were used in our analysis are included in Supplementary Material A. The first step in the workflow (see Fig. 1) was to harmonize the datasets using Maelström research guidelines for retrospective data^44^.

### Data synthesis method

#### Generative model

We used a sequential synthesis method using sequence-optimized decision trees^24^. With sequential synthesis models, a variable is synthesized by using the values earlier in the sequence as predictors. All variables used in the analysis were synthesized (step 2 of the workflow as illustrated in Fig. 1). Only the CCHS dataset was synthesized.

Sequential trees have been used to synthesize health and social sciences data^45–53^, and applied in research studies on synthetic data^45,54,55^. Additional improvements were implemented to the basic sequential synthesis method for this study. Each model in the sequence was trained using a gradient boosted decision tree^56,57^ with Bayesian optimization for hyperparameter selection^58^. Each combination of hyperparameters was selected using fivefold cross validation on the training dataset during tuning.

In the context of the synthesis of categorical variables, synthetic values are generated based on the predicted probabilities. In general, boosted trees do not output correct probabilities and these need to be calibrated, especially as the number of iterations increases^59^. In addition, for imbalanced categorical outcomes, the model is trained with larger weights for the minority class, which gives incorrect probabilities. Therefore, the predicted probabilities are adjusted using beta calibration^60^.

For each continuous variable $X_{i}$ we first convert them to a Gaussian distribution. The empirical cdf was applied to each variable $F_{i} (X_{i} )$, and then the quantile function for the standard normal was applied, $\Phi^{ - 1} (F_{i} (X_{i} ))$, which is passed through for synthesis. After synthesis, the generated values $\hat{X}_{i}$ are converted back as $F_{i}^{ - 1} (\Phi (\hat{X}_{i} ))$.

#### Combining rules for synthetic data

The original proposal for synthetic data generation treated it as a form of multiple imputation^61^. Under the multiple imputation model, multiple datasets, say *m*, are synthesized and combining rules are used to compute the parameter estimates and variances for partial synthesis across the *m* synthetic datasets^62,63^. Such corrections for the parameter estimates and variances ensured that variability introduced by the synthesis process are accounted for when making population inferences from synthetic datasets.

In the context of the current study, a partial synthesis is performed in that only the Canadian dataset is replaced with the synthetic version.

For a particular model parameter $${\text{q}}_{{\text{i}}}$$ with variance $${\text{v}}_{{\text{i}}}$$ using synthetic dataset *i* where $$i = 1 \ldots m$$. The adjustment for the model parameters and variances are as follows^51,64,65^. The combined model parameter $${\overline{\text{q}}}_{m}$$ is the mean across the *m* model parameters from the synthetic datasets $${\overline{\text{q}}}_{m} \, = \,{\raise0.7ex\hbox{$1$} \!\mathord{\left/ {\vphantom {1 m}}\right.\kern-0pt} \!\lower0.7ex\hbox{$m$}}\,\sum\limits_{i} {q_{i} }$$, and $${\overline{\text{v}}}_{m}$$ is the mean variance across the *m* model parameters from the synthetic datasets where $${\overline{\text{v}}}_{m} \; = \,{\raise0.7ex\hbox{$1$} \!\mathord{\left/ {\vphantom {1 m}}\right.\kern-0pt} \!\lower0.7ex\hbox{$m$}}\,\sum\limits_{i} {v_{i} }$$ . The between imputation variance is given by $$b_{m} = \frac{1}{m - 1}\sum\limits_{i = 1}^{m} {\left( {q_{i} - \overline{q}_{m} } \right)^{2} }$$, and the adjusted variance is computed as $$T_{p} = {\raise0.7ex\hbox{${b_{m} }$} \!\mathord{\left/ {\vphantom {{b_{m} } m}}\right.\kern-0pt} \!\lower0.7ex\hbox{$m$}} + \overline{v}_{m}$$, and the adjusted large sample 95% confidence interval of the model parameter is computed as $${\overline{\text{q}}}_{m} \, \pm \,1.96\,\sqrt {T_{f} }$$. For this study we set $$m = 10$$, which is consistent with current practice for the analysis of synthetic data^51,55,64,65^.

#### Assessing the privacy risks of the synthetic data

Privacy risk was evaluated using membership disclosure on the ten pooled synthetic datasets. The accuracy of a membership disclosure attack can be measured using the relative F1 score^30^, which indicates the ability of an adversary to correctly determine the membership status of a record. The details of the method to compute membership disclosure are provided in Supplementary Material C.

Once deemed to have low privacy risks, the synthetic dataset was sent to the Austrian team for analysis. The Austrian team pooled the source ATHIS and the synthetic CCHS datasets from both countries and built the regression models described below. This is referred to as the “pooled” dataset.

### Statistical analysis

The analysis was performed on the pooled source ATHIS data and the synthetic CCHS data (steps 4 and 5 in Fig. 1).

#### Outcome variable: cardiovascular health

Our measure of CVH was the CANHEART index. The original CANHEART index was composed from the sum of the ideal metrics for 6 cardiometabolic risk factors and behaviors including history of smoking, leisure physical activity, daily fruit and vegetable consumption, body mass index, diabetes and hypertension^32^. However, due to harmonization limitations, we had to create a modified version with available variables in both datasets. The modified CANHEART index was calculated using smoking, body mass index (BMI), diabetes and hypertension variables (see Supplementary Material B). This score ranges from 0 (worse) to 4 (best or ideal cardiovascular health).

For youth, the original CANHEART index did not include hypertension and diabetes in the score due to their low prevalence in that group. However, the index with these scores included has been validated in the juvenile population in a previous study^66^.

#### Descriptive statistics on pooled dataset

The SMD was used to statistically compare the federated and pooled datasets. SMD was selected as given our large sample size, small, clinically unimportant differences, are likely to be statistically different when using t-tests or chi squared tests. The SMD between the federated and pooled datasets was computed for each synthetic dataset generated and then averaged across all of them. An SMD greater than 0.1 is deemed as a potentially clinically important difference, a threshold often recommended for declaring imbalance in pharmacoepidemiologic research^31^.

#### Univariable and multivariable models on pooled dataset

Both univariable and multivariable linear regression models were used to determine the association between the predictors and cardiovascular health. The multivariable regression model had as predictors the following variables: sex, education level, marital status, household size, household income, immigrant status, age, and country. Goodness of fit was evaluated with R^2^ for each model.

#### Comparison between pooled partially synthetic data analysis and federated analysis

One common measure of the utility of synthetic datasets is that the data analysis results using synthetic data are similar to the analysis results using the real data (ground truth results) and that the conclusions are the same^67^. It is quite common to evaluate the utility of synthetic data generation techniques using this approach^34,35,68,69^. In our case, the ground truth results using federated analysis served as our real data results.

The utility of the pooled dataset was evaluated by comparing the pooled data regression model with the model constructed from a federated analysis which used both source datasets^25^. The federated analysis approach gives the correct results as it does not involve any distortion of the variables. The two nodes of the system were in Montreal and Vienna. A distributed analysis on the horizontally partitioned dataset was performed by exchanging interim regression results between the two nodes. Because no raw data is exchanged among the nodes the interim results sharing is not deemed to be a disclosure of personal health information (step 6 in Fig. 1).

If the pooled partially synthetic data is a good proxy for the pooled source data then we would expect the conclusions from the pooled analysis to be the same as the conclusions from federated analysis (step 7 in Fig. 1).

### Ethics

The study was approved by the research ethics boards of the McGill University Health Center (Project #2020–5452) and the Medical University of Vienna (1859/2019). All methods were carried out in accordance with relevant guidelines and regulations. Given that the datasets come from national surveys conducted by national statistical offices in each country (Statistics Canada and Statistik Austria), the respondents provided informed consent for the data collection and to the conditions for disclosing the data for further research.

## Supplementary Information

Supplementary Information.

## Data Availability

The data that support the findings of this study are available from Statistics Canada for the Canadian data and Statistik Austria for the Austrian data. However, restrictions apply to the availability of these datasets. To access the datasets, direct requests must be made to the data custodians as these are not public datasets and there may be conditions and agreements for making them available.

## Abbreviations

CVH: Cardiovascular health
CVD: Cardiovascular diseases
SDG: Synthetic data generation
US: United States
UK: United Kingdom
CCHS: Canadian community health survey
ATHIS: Austria health interview survey
BMI: Body mass index

## References

[1] Virani SS (2020). Heart disease and stroke statistics-2020 update: A report from the American Heart Association. Circulation.

[2] Huxley VH (2007). Sex and the cardiovascular system: The intriguing tale of how women and men regulate cardiovascular function differently. Adv. Physiol. Educ..

[3] Connelly PJ, Azizi Z, Alipour P, Delles C, Pilote L, Raparelli V (2021). The importance of gender to understand sex differences in cardiovascular disease. Can. J. Cardiol..

[4] Bartz D (2020). Clinical advances in sex- and gender-informed medicine to improve the health of All: A review. JAMA Intern. Med..

[5] Cirillo D (2020). Sex and gender differences and biases in artificial intelligence for biomedicine and healthcare. NPJ Digit. Med..

[6] Prosperi M, Min JS, Bian J, Modave F (2018). Big data hurdles in precision medicine and precision public health. BMC Med. Inform. Decis. Mak..

[7] van Panhuis WG (2014). A systematic review of barriers to data sharing in public health. BMC Public Health.

[8] Kalkman S, Mostert M, Gerlinger C, van Delden JJM, van Thiel GJMW (2019). Responsible data sharing in international health research: A systematic review of principles and norms. BMC Med. Ethics.

[9] T. Rabesandratana, “European data law is impeding studies on diabetes and Alzheimer’s, researchers warn,” *Science | AAAS*, Nov. 20, 2019. https://www.sciencemag.org/news/2019/11/european-data-law-impeding-studies-diabetes-and-alzheimer-s-researchers-warn (Accessed 21 June 2021).

[10] Bentzen HB, Castro R, Fears R, Griffin G, ter Meulen V, Ursin G (2021). Remove obstacles to sharing health data with researchers outside of the European Union. Nat. Med..

[11] Zhou Z (2005). Effectiveness of statins for secondary prevention in elderly patients after acute myocardial infarction: an evaluation of class effect. CMAJ.

[12] Deeks JJ, Altman DG, Bradburn MJ (2001). Statistical methods for examining heterogeneity and combining results from several studies in meta-analysis. Systematic Reviews in Health Care.

[13] Suissa S (2012). CNODES: The Canadian network for observational drug effect studies. Open Med..

[14] K. El Emam, L. Mosquera, and R. Hoptroff, *Practical Synthetic Data Generation: Balancing Privacy and the Broad Availability of Data*. Sebastopol, CA: O’Reilly Media, 2020. [Online]. https://www.oreilly.com/library/view/practical-synthetic-data/9781492072737/. Accessed 19 October 2020.

[15] El Emam K, Hoptroff R (2019). The synthetic data paradigm for using and sharing data. Cutter Executive Update.

[16] Haendel MA (2021). The National COVID Cohort Collaborative (N3C): Rationale, design, infrastructure, and deployment. J. Am. Med. Inform. Assoc..

[17] CMS, “CMS 2008–2010 Data Entrepreneurs’ Synthetic Public Use File (DE-SynPUF),” 2022. https://www.cms.gov/Research-Statistics-Data-and-Systems/Downloadable-Public-Use-Files/SynPUFs/DE_Syn_PUF (Accessed 17 July 2022).

[18] Wang Z, Myles P, Tucker A (2019). Generating and evaluating synthetic UK primary care data: Preserving data utility patient privacy. 2019 IEEE 32nd International Symposium on Computer-Based Medical Systems (CBMS).

[19] “Synthetic data at CPRD,” *Medicines & Healthcare products Regulatory Agency*, 2020. https://www.cprd.com/content/synthetic-data (Accessed 24 September 2020).

[20] NHS England, “A&E Synthetic Data.” https://data.england.nhs.uk/dataset/a-e-synthetic-data (Accessed 16 July 2022).

[21] IKNL, “Synthetic dataset,” *integraal kankercentrum Nederland*, 2021. https://iknl.nl/en/ncr/synthetic-dataset (Accessed 13 January 2023).

[22] “SNDS synthétiques,” *Systeme national des donnees de sante*, 2021. https://documentation-snds.health-data-hub.fr/formation_snds/donnees_synthetiques/ (Accessed 20 January 2022).

[23] Beata Nowok, “Utility of synthetic microdata generated using tree-based methods,” presented at the UNECE Statistical Data Confidentiality Work Session, Helsinki, Oct. 2015. [Online]. https://unece.org/statistics/events/SDC2015. Accessed 24 February 2020.

[24] El Emam K, Mosquera L, Zheng C (2021). Optimizing the synthesis of clinical trial data using sequential trees. J. Am. Med. Inform. Assoc..

[25] Wolfson M (2010). DataSHIELD: Resolving a conflict in contemporary bioscience–performing a pooled analysis of individual-level data without sharing the data. Int. J. Epidemiol..

[26] Zhang Z, Yan C, Mesa DA, Sun J, Malin BA (2020). Ensuring electronic medical record simulation through better training, modeling, and evaluation. J. Am. Med. Inform. Assoc..

[27] Zhang Z, Yan C, Lasko TA, Sun J, Malin BA (2021). SynTEG: A framework for temporal structured electronic health data simulation. J. Am. Med. Inform. Assoc..

[28] Goncalves A, Ray P, Soper B, Stevens J, Coyle L, Sales AP (2020). Generation and evaluation of synthetic patient data. BMC Med. Res. Methodol..

[29] Hilprecht B, Härterich M, Bernau D (2019). Monte Carlo and reconstruction membership inference attacks against generative models. Proc. Priv. Enhanc. Technol..

[30] El Emam K, Mosquera L, Fang X (2019). Validating a membership disclosure metric for synthetic health data. JAMIA Open.

[31] Stuart EA, Lee BK, Leacy FP (2013). Prognostic score–based balance measures for propensity score methods in comparative effectiveness research. J. Clin. Epidemiol..

[32] Maclagan LC (2014). The CANHEART health index: A tool for monitoring the cardiovascular health of the Canadian population. CMAJ.

[33] Pedhazur E (1982). Multiple Regression in Behavioral Research.

[34] Benaim AR (2020). Analyzing medical research results based on synthetic data and their relation to real data results: Systematic comparison from five observational studies. JMIR Medical Informatics.

[35] El Emam K, Mosquera L, Jonker E, Sood H (2021). Evaluating the utility of synthetic COVID-19 case data. JAMIA Open.

[36] Mosquera L (2023). A method for generating synthetic longitudinal health data. BMC Med. Res. Methodol..

[37] Kaplan RM, Kronick RG (2006). Marital status and longevity in the United States population. J. Epidemiol. Community Health.

[38] Kilpi F, Konttinen H, Silventoinen K, Martikainen P (2015). Living arrangements as determinants of myocardial infarction incidence and survival: A prospective register study of over 300,000 Finnish men and women. Soc. Sci. Med..

[39] Ikeda A (2009). Living arrangement and coronary heart disease: The JPHC study. Heart.

[40] Schultz WM (2017). Marital status and outcomes in patients with cardiovascular disease. J. Am. Heart Assoc..

[41] Dhindsa DS, Khambhati J, Schultz WM, Tahhan AS, Quyyumi AA (2020). Marital status and outcomes in patients with cardiovascular disease. Trends Cardiovasc. Med..

[42] Koskenvuo M, Kaprio J, Romo M, Langinvainio H (1981). Incidence and prognosis of ischaemic heart disease with respect to marital status and social class. A national record linkage study. J. Epidemiol. Community Health.

[43] Schaefer C, Quesenberry CP, Wi S (1995). Mortality following conjugal bereavement and the effects of a shared environment. Am. J. Epidemiol..

[44] Fortier I (2017). Maelstrom Research guidelines for rigorous retrospective data harmonization. Int. J. Epidemiol..

[45] Drechsler J, Reiter JP (2011). An empirical evaluation of easily implemented, nonparametric methods for generating synthetic datasets. Comput. Stat. Data Anal..

[46] Arslan RC, Schilling KM, Gerlach TM, Penke L (2021). Using 26,000 diary entries to show ovulatory changes in sexual desire and behavior. J. Pers. Soc. Psychol..

[47] Bonnéry D (2019). The promise and limitations of synthetic data as a strategy to expand access to state-level multi-agency longitudinal data. J. Res. Educ. Effect..

[48] Sabay A, Harris L, Bejugama V, Jaceldo-Siegl K (2018). Overcoming small data limitations in heart disease prediction by using surrogate data. SMU Data Sci. Rev..

[49] Michael Freiman, Amy Lauger, and Jerome Reiter, “Data Synthesis and Perturbation for the American Community Survey at the U.S. Census Bureau,” US Census Bureau, Working paper, 2017. [Online]. https://www.census.gov/library/working-papers/2018/adrm/formal-privacy-synthetic-data-acs.html. Accessed 24 February 2020.

[50] B. Nowok, “Utility of synthetic microdata generated using tree-based methods,” presented at the UNECE Statistical Data Confidentiality Work Session, Helsinki, Oct. 2015. [Online]. https://unece.org/statistics/events/SDC2015. Accessed 24 February, 2020.

[51] Raab GM, Nowok B, Dibben C (2016). Practical data synthesis for large samples. J. Priv. Confid..

[52] Nowok B, Raab GM, Dibben C (2017). Providing bespoke synthetic data for the UK Longitudinal Studies and other sensitive data with the synthpop package for R 1. Stat. J. IAOS.

[53] Quintana DS (2020). “A synthetic dataset primer for the biobehavioural sciences to promote reproducibility and hypothesis generation. eLife.

[54] C. Little, M. Elliot, R. Allmendinger, and S. Samani, “Generative adversarial networks for synthetic data generation: A comparative study,” presented at the UNECE Expert Meeting on Statistical Data Confidentiality, Poznań, Poland: United Nations Economic Commission for Europe, Dec. 2021, p. 17. [Online]. https://unece.org/statistics/documents/2021/12/working-documents/generative-adversarial-networks-synthetic-data. Accessed 17 January 2022.

[55] Taub J, Elliot M, Sakshaug W (2020). The impact of synthetic data generation on data utility with application to the 1991 UK samples of anonymised records. Trans. Data Priv..

[56] Bühlmann P, Hothorn T (2007). Boosting algorithms: Regularization, prediction and model fitting. Statist. Sci..

[57] G. Ke *et al.*, “LightGBM: A Highly Efficient Gradient Boosting Decision Tree,” in *Advances in Neural Information Processing Systems 30*, I. Guyon, U. V. Luxburg, S. Bengio, H. Wallach, R. Fergus, S. Vishwanathan, and R. Garnett, Eds., Curran Associates, Inc., 2017, pp. 3146–3154. [Online]. http://papers.nips.cc/paper/6907-lightgbm-a-highly-efficient-gradient-boosting-decision-tree.pdf. Accessed 15 October 2020.

[58] J. Snoek, H. Larochelle, and R. P. Adams, “Practical Bayesian optimization of machine learning algorithms,” in *Proceedings of the 25th International Conference on Neural Information Processing Systems - Volume 2*, in NIPS’12. Red Hook, NY, USA: Curran Associates Inc., 2012, pp. 2951–2959.

[59] A. Niculescu-Mizil and R. A. Caruana, “Obtaining Calibrated Probabilities from Boosting,” arXiv:1207.1403* [cs, stat]*, Jul. 2012. [Online]. http://arxiv.org/abs/1207.1403. Accessed 21 October 2020.

[60] M. Kull, T. S. Filho, and P. Flach, “Beta calibration: a well-founded and easily implemented improvement on logistic calibration for binary classifiers,” in *Proceedings of the 20th International Conference on Artificial Intelligence and Statistics*, PMLR, Apr. 2017, pp. 623–631. [Online]. https://proceedings.mlr.press/v54/kull17a.html. Accessed 30 December 2022.

[61] Rubin D (1993). Discussion: Statistical disclosure limitation. J. Off. Stat..

[62] Raghunathan T, Reiter J, Rubin D (2003). Multiple imputation for statistical disclosure control. J. Off. Stat..

[63] Reiter JP (2002). Satisfying disclosure restrictions with synthetic data sets. J. Off. Stat..

[64] Reiter J (2003). Inference for partially synthetic, public use microdata sets. Surv. Methodol..

[65] Loong B, Zaslavsky AM, He Y, Harrington DP (2013). Disclosure control using partially synthetic data for large-scale health surveys, with applications to CanCORS. Stat. Med..

[66] Azizi Z (2021). Sex, gender, and cardiovascular health in Canadian and Austrian Populations. Can. J. Cardiol..

[67] El Emam K (2020). Seven ways to evaluate the utility of synthetic data. IEEE Secur. Priv..

[68] Azizi Z, Zheng M, Mosquera L, Pilote L, El Emam K (2021). Can synthetic data be a proxy for real clinical trial data ? A validation study. BMJ Open.

[69] Beaulieu-Jones BK (2019). Privacy-preserving generative deep neural networks support clinical data sharing. Circ. Cardiovasc. Qual. Outcomes.

