# Supplementary material for: A comparison of synthetic data generation and federated analysis for enabling international evaluations of cardiovascular health
Source: Sci Rep. 2023 Jul 17;13:11540. doi: 10.1038/s41598-023-38457-3 (PMC10352377; doi:10.1038/s41598-023-38457-3)
Supplement: Supplementary file 1 — Supplementary Information. [file 41598_2023_38457_MOESM1_ESM.docx]

# Supplementary Materials

## Supplementary Materials A: CCHS Definition of Variables

The following were the CCHS variables that were used on our study.

|  | **CCHS** | | **ATHIS** | | **Harmonization** | |
| --- | --- | --- | --- | --- | --- | --- |
| **Variables** | **Question Wording** | **Response Categories** | **Question Wording** | **Response Categories** | **Categories** | **Definition** |
| Sex:  Female  Male | Enter the respondent's sex. If necessary, ask: Is respondent male or female? | 1 Male  2 Female | Sex | 1 Male  2 Female | 1,2 | 1 Male  2 Female |
| Age:  <20  20-29  30-39  40-49  50-59  60-69  >=70 | What is your age? | 12 To 14 Years  15 To 17 Years  18 To 19 Years  20 To 24 Years  25 To 29 Years  30 To 34 Years  35 To 39 Years  40 To 44 Years  45 To 49 Years  50 To 54 Years  55 To 59 Years  60 To 64 Years  65 To 69 Years  70 To 74 Years  75 To 79 Years  80 Years Or More | Age (5 year groups) | 1= 15-19  2= 20-24  3= 25-29  4= 30-34  5= 35-39  6= 40-44  7= 45-49  8= 50-54  9= 55-59  10= 60-64  11= 65-69  12=70-74  13= 75-79  14= 80-84  15= 85+ | 1,2,3,4,5,6,7 | 1:"<20"  2:"20-29"  3:"30-39" 4:"40-49"  5:"50-59"  6:"60-69"  7:"=>70" |
| Immigrant | Immigrant | Yes  No | Immigration In which country have you been born? | 1. Austria 2. Other (name the country) | 0,1 | 0=No  1=Yes |
| Marital Status:  Single  Divorced/widowed  Common-law/married | What is your marital status? Are you married, living common-law, widowed, separated, divorced, or single, never married? | Married  Common-Law  Widow/Sep/Div  Single/Never Mar | What is your legal marital status? | 1. Single  2. married or registered partnership and living together  3. married or registered partnership and living apart  4. widow  5. divorced  6. no statement | 1,2,3 | 1=single/never married, 2=widow/separated/divorced, 3=common in law/married |
| Household Size:  1  2  3  4  5 & 5+ | Household size | 1 Person  2 Persons  3 Persons  4 Persons  5 Or + Persons |  | 1 Person  2 Persons  3 Persons  4 Persons  5 Or + Persons | 1,2,3,4,5 | 1 Person  2 Persons  3 Persons  4 Persons  5 Or + Persons |
| Education  <Secondary  Secondary  Post secondary  >Post secondary | Highest level of education - respondent, 4 levels | < Than Secondary  Secondary Grad  Post-Sec  Post-Sec. Grad | Highest completed education? | 1. Primary education  2. Lower secondary  3. upper secondary  4. Post-secondary non-tertiary education  5. Short-cycle tertiary education  6. Bachelor's or equivalent  7. Master's or equivalent  8. Doctoral or equivalent | 1,2,3,4 | CA: 1= Less than secondary, 2= Secondary, 3= Post-secondary education, 4= Post-secondary grad degree  AT:  1="ISCED 1", "ISCED 2 (inkl BMS<2J)"  2="ISCED 3 (Zugangzu ISCED 5/6/7)"  3="ISCED 4", "ISCED 5"  3="ISCED 6", "ISCED 7", "ISCED 8" |
| BMI  <25 | BMI / self-report | Based On Weight (Kilograms) / Self-Reported And Height (Metres) / Self-Reported | Height without shoes  Weight without clothes and shoes | …cm  ….Kg | 0,1 | 0:<25 , 1:>=25 |
| HX smoking  Current daily, occasional smoking | Type of smoker | Daily  Occasional  Always Occasion  Former Daily  Former Occasion.  Never Smoked | Current Smoker Do you smoke? | 1. Yes, sometimes 2. Yes, daily 3. no | 0,1 | 1=Former or none smoker, 0=Current |
| Household Income  Low (NO OR <$20K)  Medium ($20K- $60K)  High (>$60K) | Total household income from all sources | No Or <$20,000  $20,000-$39,999  $40,000-$59,999  $60,000-$79,999  $80,000 Or More | Household Income  How much does your household earn per month after taxes? | 1. Up to 600€ 2. 601-900€ 3. 901-1200€ 4. 1201-1500€ 5. 1501-1800€ 6. 1801-2200€ 7. 2201-2600€ 8. 2601-3000€ 9. 3001-3500€ 10. 3501-4000€ 11. 4001-4500€ 12. 4501-5000€ 13. 5001-6000€ 14. 6001-8000€ 15. 8001 and more | 3,2,1 | 3: Low (CA: <20K)  2: Med (CA:20K-60K)  1: High (CA: >60K)  3:Low (AT:"Unter dem 1. Quintil" |HHINCOMEf =="Zwischen dem 1. und dem 2. Quintil")  2: Med (AT:"Zwischen dem 2. und dem 3. Quintil")  1: High(AT:"Zwischen dem 3. und dem 4. Quintil" ,"Zwischen dem 4 und dem 5. Quintil") |
| HX Hypertension | Remember, we’re interested in conditions diagnosed by a health professional and are expected to last or have already lasted 6  months or more. Do you have high blood pressure? | Yes  No | Hypertension Did you have hypertension in the last 12 months? | 1. Yes 2. no | 0,1 | 0=No  1=Yes |
| HX Diabetes | (Remember, we’re interested in conditions diagnosed by a health professional and that are expected to last or have already  lasted 6 months or more.)  Do you have diabetes? | Yes  No | Diabetes  Did you have diabetes in the last 12 months? | 1. Yes 2. no | 0,1 | 0=No  1=Yes |

## Supplementary Materials B: Modified CANHEART Score

Modified CANHEART index definition in both Canadian and Austrian populations.

|  | **CANHEART:**  **Canadian population** | **ATHEART:**  **Austrian population** |
| --- | --- | --- |
| **Smoking** | Non-Smoker or former daily or occasional smoker who quit more than 12 months | Non-Smoker or former daily or occasional smoker who quit more than 12 months |
| **Overweight/obesity** | BMI<25 | BMI<25 |
| **Hypertension** | No self-reported HTN diagnosed by health professional | No self-reported HTN diagnosed by health professional |
| **Diabetes** | No self-reported diabetes diagnosed by health professional | No self-reported diabetes diagnosed by health professional |

## Supplementary Materials C: Method for Computing Membership Disclosure

The starting assumption for computing membership disclosure is that the synthetic data distribution approximates the real dataset distribution [1]. Therefore, the probability that an adversary’s dataset belongs to the training dataset is proportional to the probability that the attack dataset belongs to the synthetic dataset. This is assessed through the minimal Hamming distance between an adversary record and synthetic records [2]. Hamming distance is a measure of the number of values that differ between the two sets of real and synthetic variables. If the minimal distance is below a threshold distance then the attack record is said to match a synthetic record. A threshold distance value for the of 5 was used, which is at the high end used in the literature based on a recent review [1].

The baseline membership disclosure method is illustrated in figure below. Here the real dataset is randomly split into two subsets, the training sample, and a holdout sample. The training sample is then synthesized, and a synthetic dataset is created. We will call this synthetic dataset $S$.

We assume that an adversary has some information on $w$ patients, where say $w\times t$ are drawn from the training sample and $w\times\left(1-t\right)$ are drawn from the holdout sample, where $t$ is some fraction. For example, if $t=0.5$ then the attack dataset is half training and half holdout. We will denote this attack dataset as $D$, and we have $w=\left|D\right|$. The adversary would not know whether a record in $D$ was drawn from the training sample or from the holdout sample and would not know the proportion of records which came from the training sample out of the $w$. We set$w=s\times n$ were $s$ is a sampling fraction, and$n$ is the size of the real dataset.

Previous work did not demonstrate a pronounced change in multiple membership disclosure metrics when the sampling fraction was altered [4], [5]. Therefore, we will not consider $s$ to be a key parameter.

We can then compute the minimum distance between every record in $D$ and all the records in synthetic dataset $s$. In the literature, the distance is measured using the Hamming distance, and a match for an attack record is considered to have occurred if the minimal distance across all real records i.e. below the threshold. The accuracy of that assessment can be evaluated using the F1 score.


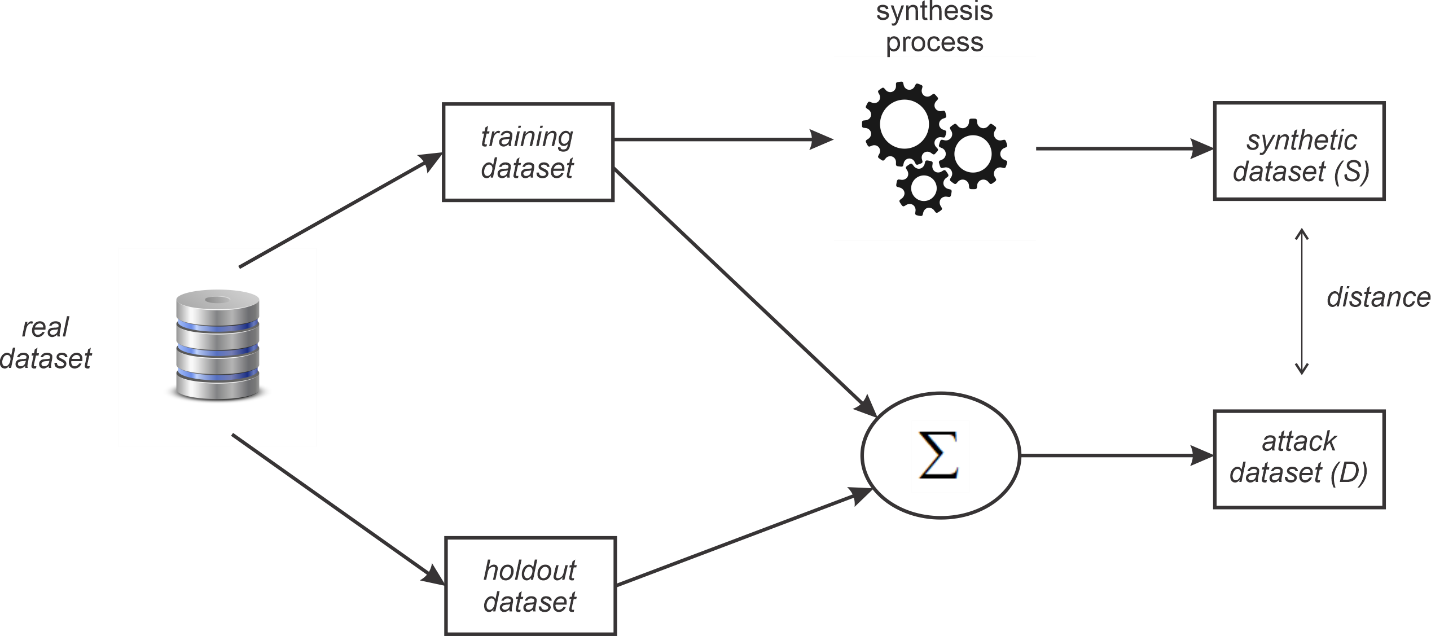


An overview of the membership disclosure evaluation process that is commonly used in the literature.

**Supplementary Materials D: Individual Country Model**

| CANHEART score** | CCHS-Source | ATHIS-Source |
| --- | --- | --- |
|  | **Coefficient (β)***** | **Coefficient (β)***** |
| Sex (Female)  Education (*ref:*<secondary)  Marital status (*ref:* Single)  Divorced widowed  Married  Household Size (*ref:* 1person)  House Income (*ref:* High)  Immigrant  Age *(ref: <20)*  Country (*ref:*CA) | 0.22(0.21, 0.24)  0.03(0.03, 0.04)  -0.11(-0.13, -0.08)  -0.16(-0.19, -0.14)  0.05(0.04, 0.06)  -0.13(-0.14, -0.12)  0.16 (0.14, 0.18)  -0.12 (-0.13, -0.12)  - | 0.38(0.35,0.41)  0.12(0.10,0.14)  -0.15(-0.21,-0.09)  -0.11(-0.16,-0.06)  0.04(0.02,0.06)  -0.008(-0.03,0.01)  -0.04(-0.10,0.01)  -0.19(-0.20, -0.17) |
| ***CANHEART index: A measure of CVH in the population, consisting of 4 cardiometabolic risk factors (i.e. smoking, obesity, diabetes and hypertension), 0 (worst) to 4(ideal)*  **** β Coefficient: the degree of change in the CANHEART index for every 1-unit of change in the predictor variables* | | |

**Table D.1:** Country specific main effects multivariable regression model.

**References**

[1] H. Sun, T. Zhu, Z. Zhang, D. Jin, P. Xiong, and W. Zhou, “Adversarial Attacks Against Deep Generative Models on Data: A Survey,” *IEEE Trans. Knowl. Data Eng.*, no. 01, pp. 1–1, Nov. 2021, doi: 10.1109/TKDE.2021.3130903.

[2] “Hamming Distance - an overview | ScienceDirect Topics.” https://www.sciencedirect.com/topics/engineering/hamming-distance (accessed Mar. 26, 2022).

[3] O. Mendelevitch and M. D. Lesh, “Fidelity and Privacy of Synthetic Medical Data,” *ArXiv210108658 Cs*, Jun. 2021, Accessed: Jul. 05, 2021. [Online]. Available: http://arxiv.org/abs/2101.08658

[4] E. Choi, S. Biswal, B. Malin, J. Duke, W. F. Stewart, and J. Sun, “Generating Multi-label Discrete Patient Records using Generative Adversarial Networks,” in *Proceedings of Machine Learning for Healthcare 2017*, MLResearchPress, 2017, pp. 286–305. Accessed: Jul. 11, 2019. [Online]. Available: http://proceedings.mlr.press/v68/choi17a/choi17a.pdf

[5] Z. Zhang, C. Yan, D. A. Mesa, J. Sun, and B. A. Malin, “Ensuring electronic medical record simulation through better training, modeling, and evaluation,” *J. Am. Med. Inform. Assoc.*, doi: 10.1093/jamia/ocz161.

[6] C. Yan, Z. Zhang, S. Nyemba, and B. A. Malin, “Generating Electronic Health Records with Multiple Data Types and Constraints,” *ArXiv200307904 Cs Stat*, Mar. 2020, Accessed: Jun. 28, 2020. [Online]. Available: http://arxiv.org/abs/2003.07904

[7] A. Goncalves, P. Ray, B. Soper, J. Stevens, L. Coyle, and A. P. Sales, “Generation and evaluation of synthetic patient data,” *BMC Med. Res. Methodol.*, vol. 20, no. 1, p. 108, Dec. 2020, doi: 10.1186/s12874-020-00977-1.
